# Comparison and Evaluation of Attitude and Knowledge Towards the Management of Dental Injury in School Teachers Before and After Oral Health Education

**DOI:** 10.5005/jp-journals-10005-1551

**Published:** 2018-10-01

**Authors:** Neha Nashine, Arpana Bansal, Parimala Tyagi, Manish Jain, Ankur Jain, Utkarsh Tiwari

**Affiliations:** 1 MDS Student, Department of Pedodontics and Preventive Dentistry, Peoples Dental Academy, Madhya Pradesh, India; 2 Professor, Department of Pedodontics and Preventive Dentistry, Peoples Dental Academy, Madhya Pradesh, India; 3 Professor and HOD, Department of Pedodontics and Preventive Dentistry, Peoples Dental Academy, Madhya Pradesh, India; 4 Reader, Department of Community and Preventive Dentistry, Peoples Dental Academy, Madhya Pradesh, India; 5 Reader, Department of Pedodontics and Preventive Dentistry, Peoples Dental Academy, Madhya Pradesh, India; 6 Senior Lecturer, Department of Pedodontics and Preventive Dentistry, Peoples Dental Academy, Madhya Pradesh, India

**Keywords:** Attitude, Avulsed tooth, Dental trauma, Interventional study, Knowledge, School teachers.

## Abstract

**Background:**

Dental traumatic injuries are prevalent in school-going children. Therefore, It is critical to ascertain the knowledge and practices of school teachers who are in close contact with these children.

**Aim:**

To evaluate the knowledge and attitude of school teachers toward dental trauma and the effect of the educational intervention.

**Materials and methods:**

The interventional study was designed by two-stage cluster sampling. A total of 158 teachers were part of the research. Data was collected through a pretested questionnaire. Re-evaluation was done after an informative lecture. Results were statistically analyzed.

**Result:**

The knowledge was consistently lacking prior to intervention with the level of correct answer ranging from 0.6 to 56.3%. It improved significantly post education ranging up to 96.6%. A positive attitude was noticed even before the intervention was employed.

**Conclusion:**

Study observes a significant knowledge gap regarding the primary management of dental injuries amongst school teachers and emphasizes the need for urgent educational intervention to fill in this gap.

**Clinical significance:**

Suggestions derived from this study are especially important in today’s scenario where, the concept of conservation, retention, prevention and early intervention of tooth structures is topmost of all the priorities.

**How to cite this article:** Nashine N, Bansal A, Tyagi P, Jain M, Jain A, Tiwari U. Comparison and Evaluation of Attitude and Knowledge towards the Management of Dental Injury in School Teachers Before and After Oral Health Education. Int J Clin Pediatr Dent. 2018;11(5):425-429.

## INTRODUCTION

Worldwide dental trauma is a significant pediatric oral health issue. Traumatic dental injuries are responsible for a considerable portion of dental health emergencies, requiring multiple follow-up visits and also leading to long-term consequences for the developing dentition. Furthermore, dental trauma can lead to psychological, social and financial challenges for children, parents and health authorities especially in the developing world.^1^

Multiple studies have demonstrated that 20% of the permanent teeth of 7 to 11-year-old children had been involved in some traumatic accident. Most of which involve anterior teeth.^2^ Statistics reveal that dental avulsion injuries are common in children because of the intensity and frequency of the trauma as well as the inclination of the teeth in the oral cavity in children of this age group.^3^

The two most common locations where traumatic dental injuries occur are home and school.^4^ Children suffer accidental injuries due to their play activities such as running, skating, bike riding, etc. at home as well as in school.^5^

A child spends maximum time in school after his home. Over 16% of total dental injuries occur in the school environment, and 19% of the injuries occur due to falling.^6^ A teacher is a primary caretaker and mentor during this time.^5^ School-going children in India spends approximately 6 to 7 hours daily in school which is approximately one-fourth time of the day.^7^ For this reason, the participation of school teacher in the emergency situation including dental emergencies is important to provide care to the injured child. The quality of dental emergency management will directly affect the long-term prognosis of the tooth.^5^ Optimal emergency response for traumatized teeth is critical for ultimate treatment success. Therefore, it is critical to ascertain the knowledge and practice of school teacher in school who are in close contact with the young individual.^8^

Various studies in western countries have revealed that the teachers have poor knowledge regarding dental trauma.^2,4,5,8^ In India, there have been very few studies conducted to evaluate the knowledge and attitude of school teacher regarding dental trauma.^3^ ^6^ ^9^ ^10^ A small number of studies which were done showed inadequate knowledge in managing traumatic injuries in children in southern India.^6,9^

So, a study was designed to evaluate knowledge and attitude of school teacher toward traumatic injuries in Central India. Concomitantly, different measures were taken to impart knowledge regarding dental trauma and its management following which the knowledge and attitude were re-evaluated.

## MATERIALS AND METHODS

This study was interventional and was conducted among 158 school teachers in Bhopal city, Madhya Pradesh, India. Two-stage cluster sampling was used for the selection of subjects. In the first stage, two out of four zones in the city were selected. All the government schools in the selected zones were listed out. Using this as a sampling frame, 10% of the schools in these zones were chosen subsequently using a random sampling technique. The teachers engaged in teaching children aged 8 to 11 years in these selected schools who agreed to participate in the research after a written consent were included in the study.

The data collection was done by a single trained investigator to avoid inter-examiner variability. A survey proforma and questionnaire with closed-ended questions was used to collect information on demography, first aids, management of dental trauma and transport of the tooth. The teachers were then educated through audio and visual aids regarding management of dental trauma and again data collected through the same questionnaire for post-intervention analysis. Data comparison was done by applying specific statistical tests with the help of the Statistical Package for Social Science (SPSS Version 20; Chicago Inc., USA). A p-value of less than 0.05 was considered statistically significant.

## RESULT

**Graphs 1A and B:**
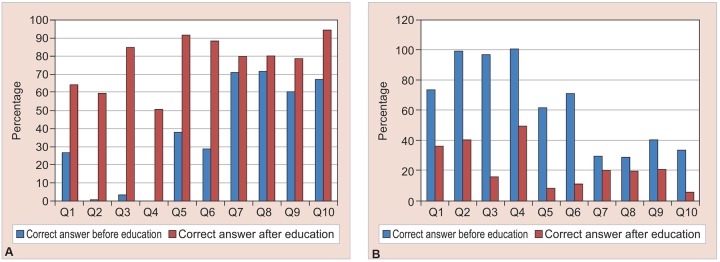
(A) Showing the increase in percentage of correct answers after educational intervention; (B) Showing the decrease in percentage of wrong answers after educational intervention

Table 1 and Graphs 1A and 1B shows the knowledge and attitude of teachers prior to the educational intervention, and also compares it after the educational intervention. The understanding was consistently lacking in almost all questions prior to the invasion. The level of correct answer varied from 0.6 to 56.3%. In contrast, the attitude was seen to be more positive, and most teachers wanted to learn proper management skill for handling the dental traumatic injuries. No significant differences were found in knowledge and attitude score concerning teachers work experience and gender (p > 0.05).

**Table 1:** **Table 1:** Comparison of knowledge and attitude before and after dental health education

		Before education				After education			
Question number		*Correct answer*		*Wrong answer*		*Correct answer*		*Wrong answer*	
Q1		26.6%		73.4%		63.9%		36.1%	
Q2		0.6%		99.4%		59.5%		40.5%	
Q3		3.2%		96.8%		84.8%		15.2%	
Q4		0%		100%		50.6%		49.4%	
Q5		38%		62%		91.8%		8.2%	
Q6		29.1%		70.9%		88.6%		11.4%	
Q7		70.9%		29.1%		79.7%		20.3%	
Q8		71.5%		28.5%		80.4%		19.6%	
Q9		60.1%		39.9%		79.1%		20.9%	
Q10		67.1%		32.9%		94.3%		5.7%	

After the health education, significant improvement was observed in knowledge about the correct management of dental injuries. The level of correct answers increased significantly which varied from 59.5 to 96.7%. Improvement in positive attitude was also seen in teachers after receiving the health education.

**Graph 2:**
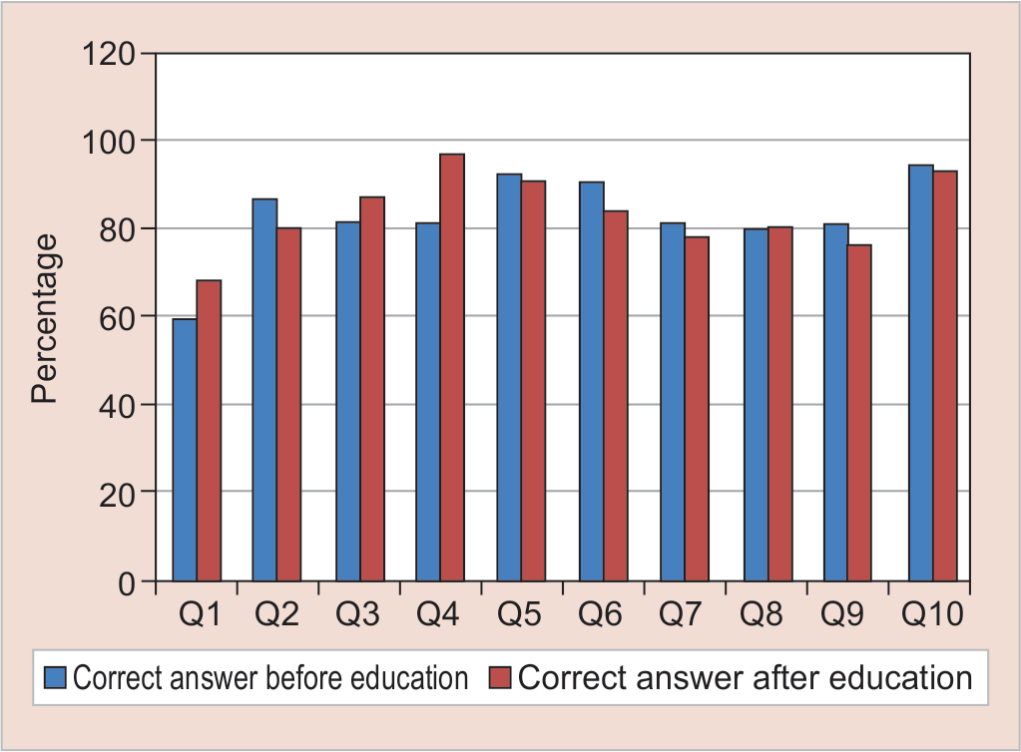
Showing comparison of two aids used in educational intervention

Graph 2 shows the comparison of audio vs. audiovisual intervention. No statistically significant difference was found in the improvement of knowledge with regards to these two aids of intervention.

## DISCUSSION

The incidence of dental injuries in children is extremely high. Most of these occur at home followed by a school environment. The current management of dental traumatic injuries aims at preventing the loss of traumatized teeth because of inaccurate diagnosis and improper treatment in pre-teenage children. In school going children, teachers are likely to be in contact with the child soon after the injury. It is their knowledge of emergency dental procedures, which is crucial to ensure a better outcome of the clinical treatment.^9^

Many studies all over the world were conducted to evaluate the knowledge of teachers towards dental trauma. Various researchers from Singapore,^11^ Hong Kong,^12^ Brazil^5^ and Italian^13^ found a lack of knowledge regarding the subject among teachers. Studies from the different part of India in Bengaluru,^6^ Himachal Pradesh^3^ and Ahmednagar^9^ concluded a similar result in their studies. In the present study, we observed a deficiency in knowledge of teachers about the management of dental trauma which is similar to national and international data.

The first part of the questionnaire was on the general management of traumatic tooth. In the present study, only 29.7% of teachers had knowledge about the general management of dental trauma. A Bengaluru based study by Mohandas et al.^16^ revealed that only 27.5% of physical education teachers knew about the management of dental injuries. Similar questionnaire-based studies conducted among school teachers by Raoof et al.,^10^ Sae-Lim et al.^11^ and Young et al.^12^ showed that 94.6%, 63% and 67.8% of total teachers respectively were having inadequate knowledge regarding the subject.

The second part of the questionnaire in the present study was related to management of avulsed tooth. It was observed that knowledge of school teachers regarding management of avulsed tooth was poor 0.6 to 3.2%. These results were comparable to studies by Roof et al.^10^ and Hasim et al.^14^ where very few teachers (i.e., 0% and 13% respectively) had knowledge about management of avulsed teeth. In the present study, the knowledge for management of fractured tooth was better (26.6%) as against that of the avulsed tooth (3.2%). This could be due to a general belief that avulsed teeth are non-salvageable. Surprisingly more teachers (38%) were having correct knowledge about the storage medium of the tooth in contrast to studies by Hashim et al.^14^ (11.2%) and Toure et al.^15^ (24.74%).

The third part of the questionnaire was about the general knowledge regarding trauma. In the present study, only 29.1% of the teachers knew the subject. Raoof et al10 obtained a similar result (39.3%) in his Brazil-based study.

The present study observed a knowledge gap in all three parts of the questionnaire. Even the past studies worldwide including those in India demonstrated insufficient knowledge of first-aid care for dental injuries amongst teachers.^5-7,10-12^ The lack of knowledge may be due to inadequacy or complete lack of training for the management of dental traumatic injuries amongst school teachers. Non-recognition of the importance of oral health education in the curriculum of teachers is a more significant concern.

The last part of the questionnaire in the present study was attributed to determine the attitude of the teachers towards the dental trauma. A significant number of teachers had a positive attitude towards dental trauma even before the intervention. 86.2% agreed that dental trauma is an emergency situation and 60.1% recognized the importance of teacher’s intervention in tooth survival. 70.9 % emphasized that dental trauma management should be an educational priority for teachers. When the attitude was compared with work experience there was no statistical difference. The teachers with more work experience had a similar positive attitude compared to teachers with less work experience, and they were as keen to learn as the younger generation. To the best of our knowledge, this type of comparison has not been done in any previous studies. An Iran based study by Raoof et al.^10^ to evaluate the attitude of the school teachers showed that 55.5% of teachers felt responsible for the provision of emergency care to the dental trauma suffered by their students. Indian studies by Mohandas et al.^6^ in Bengaluru observed that 79.1% of teachers had a positive attitude toward the importance and their role in the management of dental emergencies and trauma.

To see the impact of an educational intervention, an informative lecture was conducted for study participants, using two different methods of teaching, audio and audiovisual. To our fullest knowledge, no Indian study has been conducted in the past with this type of pre and post intervention framework. Statistically significant increase in knowledge of study subjects was observed after the informative lecture. In some questions, the knowledge has increased from 3.2 to 84.8%. Similar results were observed in a study conducted by Al-Asfour et al.^16^ in Iran with a substantial increase in the knowledge of school teachers from 39 to 97%, post-intervention.

The present study also compared the mode of imparting knowledge, i.e., audio and audiovisual aids. This is the first study in India comparing this variable. In the majority of the questions, no significant difference was found between the two groups. However, it was observed that for the ease of presenter and audience, audiovisual aid is more acceptable.

## CONCLUSION

The present study depicts the rudimentary knowledge regarding emergency management of dental injuries among school teachers and highlights the importance of educational intervention in them. More interventional studies are required on a larger scale, to assess knowledge and attitude of teachers toward the dental trauma in different parts of the country so that knowledge gap can be identified and necessary interventions can be employed. There is an urgent need to introduce and validate interventional programmes in teacher’s curriculum to sensitize them toward dental trauma and make school environment healthier.

Basic dental health education programmes for parents, school teachers will significantly reduce the undesirable sequel of traumatic injuries. This is especially important in today’s scenario where the concept of conservation, retention, and prevention of tooth structures is topmost of all the priorities. Improvement in the physical environment, closer supervision of children and adoption of health safety policies are likely to have a positive impact on prevention and better outcome of traumatic dental injuries.

### What is new in this study?

The impact of educational intervention on knowledge of school teachers was studied for the first time in India. Effect of work experience on the knowledge and attitude of teachers was compared. The present study also examined the mode of imparting knowledge, i.e. audio and audiovisual aids for the first time.
